# Influences of radiographic silicosis and drug supervisor on the development of multi drug resistant-tuberculosis in West Java, Indonesia

**DOI:** 10.1265/ehpm.24-00169

**Published:** 2025-03-20

**Authors:** Leli Hesti Indriyati, Masamitsu Eitoku, Naw Awn J-P, Miki Nishimori, Norihiko Hamada, Neni Sawitri, Narufumi Suganuma

**Affiliations:** 1 Department of Environmental Medicine, Kochi Medical School, Kochi University, Kochi, Japan; 2 Department of Occupational Health, Faculty of Medicine, University of Muhammadiyah Prof. Dr. HAMKA, Jakarta, Indonesia; 3 Department of Diagnostic and Interventional Radiology, Kochi Medical School, Kochi University, Kochi, Japan; 4 Department of Radiology, Aki General Hospital, Kochi, Japan; 5 Department of Pulmonology, RS Paru Dr.M.Goenawan Partowidigdo (RSPG), Bogor, Indonesia

**Keywords:** Silicosis, Tuberculosis, Silico-tuberculosis, MDR-TB, DOT, Indonesia

## Abstract

**Background:**

Indonesia is among countries with a high incidence of multi drug-resistant tuberculosis (MDR-TB) globally. In this study, we aim to determine the prevalence of silico-tuberculosis among TB patients and to investigate the association of radiographic silicosis and the role of drug supervisor as well as other socio-clinical factors, in the development of MDR-TB in Indonesia.

**Methods:**

A hospital-based study in West Java among 148 MDR-TB patients (case) and 164 drug-sensitive/DS-TB patients (control) was conducted. Chest x-rays were evaluated by two radiologists and one NIOSH B reader according to the ILO Classification. Face-to-face interviews were conducted using structured questionnaires to collect patients’ information, including the task of drug supervisor.

**Results:**

Findings indicate that supportive drug supervisor reduces the risk of developing MDR-TB, but silicosis showed no significant association. Nevertheless, in this study we found that 17 cases (5.4%) had silico-tuberculosis mostly exhibited as ILO profusion 3; predominated by q shape, 52.9% with large opacities and dominated by size A. Other factors significantly associated with the risk of developing MDR-TB were marital status, low income, longer traveling time to hospital, unsuccessful previous treatment and suffering drug side effects.

**Conclusion:**

This study reveals that one of preventive healthcare strategy to protect TB patients from developing MDR-TB is supportive drug supervisor. While, the development of MDR-TB was not significantly influenced by silicosis; however, there is a notable prevalence of silicosis as determined by chest radiography, highlighting the critical need for dust control, occupational hygiene, and health screening for high-risk populations.

**Supplementary information:**

The online version contains supplementary material available at https://doi.org/10.1265/ehpm.24-00169.

## Graphical Abstract

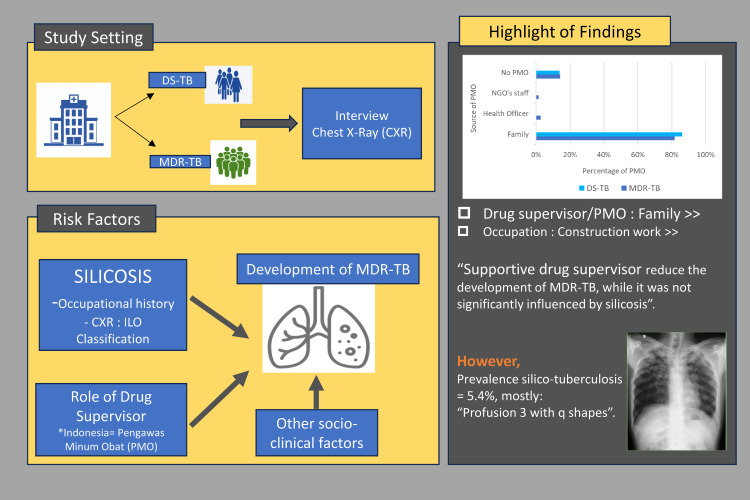

## Introduction

Indonesia is one of the countries having a significant incidence of multidrug-resistant tuberculosis (MDR-TB), with nearly half of new and relapsed cases reported among economically productive individuals aged 15 to 44 years in 2018 [1]. There is a possibility of contracting TB and transmitting it at work [2], including at worksites that generate silica dust. Occupational exposure to silica dust and silicosis has been linked to the incidence of TB in some studies [3, 4]. Yet, there is a lack of available data on the association of silicosis with the development of MDR-TB.

Patients with silico-tuberculosis are four times more likely to develop drug-resistant TB [5], with several mechanisms such as triggers of oxidative stress that interact with immunological dysfunction, leading to the survival of *Mycobacterium tuberculosis* in the lungs’ alveoli and causing resistance to the bactericidal activity of anti-TB drugs [6]. Although there is no official data on dust exposure measurement and the burden of silicosis, Indonesia has a wide range of industries at risk of silica dust exposure. Indonesia’s presidential decree on TB prevention mentions various risk factors as goals for controlling both clinical and social determinants [1]; however, workplace risk factors such as silica exposure are not addressed. Meanwhile, the August 2023 National Labor Force Survey predict that Indonesia’s working-age population in 2040 is projected to be around 211.62 million people [7].

Several studies revealed that a number of factors such as previous treatment, side effects, diabetes mellitus and treatment adherence to be associated with increased risk of developing MDR-TB [8–11]. To improve treatment adherence, the World Health Organization recommended *Directly Observed Treatment Short Course (DOTS)*, which includes Directly Observed Therapy (DOT) as the key element of DOTS. The DOT requires a supervisor to closely monitor patient compliance with medication administration [12]. This supervisor is known as Pengawas Minum Obat (PMO) in Indonesia. Prior research has investigated the effects of DOT with the development of MDR-TB, yet the results of these studies have been inconsistent [13, 14]. Some studies found that PMO has a vital role in the success of TB treatment [9, 10, 15].

However, the Ministry of Health of Indonesia discovered that, in addition to the drugs and patient characteristics, the absence of PMO or lack of monitoring from PMO is a critical element in TB treatment failure [16]. A study by Murtiwi also indicated that the presence of PMO on treatment compliance of TB patients was not effective [17]. This could be one of the reasons for high MDR-TB incidence. Although the DOTS strategy was implemented in Indonesia beginning in 1995, yet the treatment outcome for new and relapsed TB in recent years has been consistently below the global target of 90% treatment success [1].

In this study, we aim to determine the prevalence of silico-tuberculosis among TB patients from referral-to-hospital for lung disease, and (2) to investigate the association of radiographic silicosis and the role of PMO, as well as other socio-clinical factors, with MDR-TB in Indonesia. We hypothesized that TB patients with silicosis and supervised treatment by unsupportive PMO are at risk of developing MDR-TB.

## Materials and methods

### Study setting and design

This retrospective case-controlled study was conducted from March to April 2022 at RS Paru Dr.M.Goenawan Partowidigdo (RSPG), a referral hospital for lung disease in West Java, Indonesia. Our study concentrated on West Java due to its highest incidence of tuberculosis among all Indonesian provinces [1].

The inclusion criteria were as follows: (1) males and females were over the age of 25; the subjects’ cut-off age of 25 years was chosen based on the assumption that the working age in Indonesia begins at 15 years old and that the average latency period for developing chronic silicosis is around 10 years [3], implying that the youngest occupational silicosis develops around the age of 25 years; (2) availability of drug susceptibility testing (DST); and (3) availability of a chest X-ray (CXR). We defined an “MDR-TB case” as TB caused by strains of *Mycobacterium tuberculosis* which are resistant to at least Isoniazid (INH) and Rifampicin (RIF), with or without other first-line TB drugs. The term “control” refers to a TB patient who has undergone the same test and been confirmed to be drug sensitive (DS-TB). Drug susceptibility was diagnosed using the GeneXpert MTB/RIF test from sputum samples. Three hundred fifty-one (351) TB patients, aged >25 years from both sexes were enrolled in this study after providing written informed consent. Of these, patients with incomplete medical records, absence of CXR and/or mixed infection with non-TB mycobacteria were excluded.

### Data collection tool (instrument) and procedure

Face-to-face interviews were conducted using structured two-part questionnaires to collect patients’ information: (1) the first section collected information about patients’ socio-demographic, clinical data, and job history; (2) the second section collected data about the PMO’s tasks. The questionnaire on PMO’s tasks has already been validated [15] and it has 15 questions (Supplementary Table 1). The role of the PMO was classified as supportive if the overall score was 8 or higher, and less supportive if the total score was 7 or lower. Patients’ occupations were coded according to the five-digit Indonesian Standard Industrial Classification (IndSIC) 2020 [18]. Based on the literature [3, 19] possible silica exposure was identified from the reported occupation.

### Chest X-ray reading

Chest x-rays (CXR) were collected from all patients. Participants’ anonymity was protected by eliminating personal identifiers. Two independent radiologists and one NIOSH B reader determined the presence of silicosis on CXR. Final decisions in cases of disagreement were made by consensus with the B-reader (NSu). Multinodular opacities on both sides, with or without progressive massive fibrosis, and a threshold profusion of 1 in accordance with International Labour Organization (ILO) film standards, was used to diagnose silicosis [20]. The terms of the ILO 2011 classification such as profusion, small rounded opacites (p, q, r), small irregular opacities (s, t, u), large opacities (A, B, C) and others are explained in Supplementary Table 2. To increase specificity, we classified silicosis identified in CXR into one of four categories: “definite”, “probable”, “possible”, and “none”. In practice, we applied “definite” to small rounded opacites with diffuse, bilateral distribution, continuing from upper to lower; “probable” to small, rounded opacity with diffuse bilateral distribution, but not continuing from upper to middle/lower; “possible” if there was focal distribution of small rounded opacities and bilateral distribution; and “none” for CXRs that were clear, and had no small rounded opacities. The existence of silicosis was defined as “definite” or “probable.”

### Ethical considerations

The Ethics Committee for Research, Faculty of Medicine, University of Muhammadiyah Prof.Dr.HAMKA, Jakarta, Indonesia (KEPKK/FK/026/01/2022) and the Ethics Committee of Kochi Medical School (approval number: 2023-124) have approved this study. All individuals gave their informed consent to participate in the study.

### Statistical analysis

The association between the risk factors and MDR-TB, which is main result of our study, was determined by logistic regression analyses. The results were reported as odds ratios (OR) with 95% confidence interval (CI). For all statistical analysis, a *p* value of <0.05 was considered significant. The initial analysis involved descriptive statistics to summarize the sociodemographic and clinical characteristics of patients, utilizing frequencies and percentages. We employed the Chi-Square test for bivariate analysis to evaluate the associations between the categorical variable and MDR-TB. The statistical analyses were performed using Stata version 17 software (StataCorp, College Station, TX, USA).

## Results

**Fig. 1:**
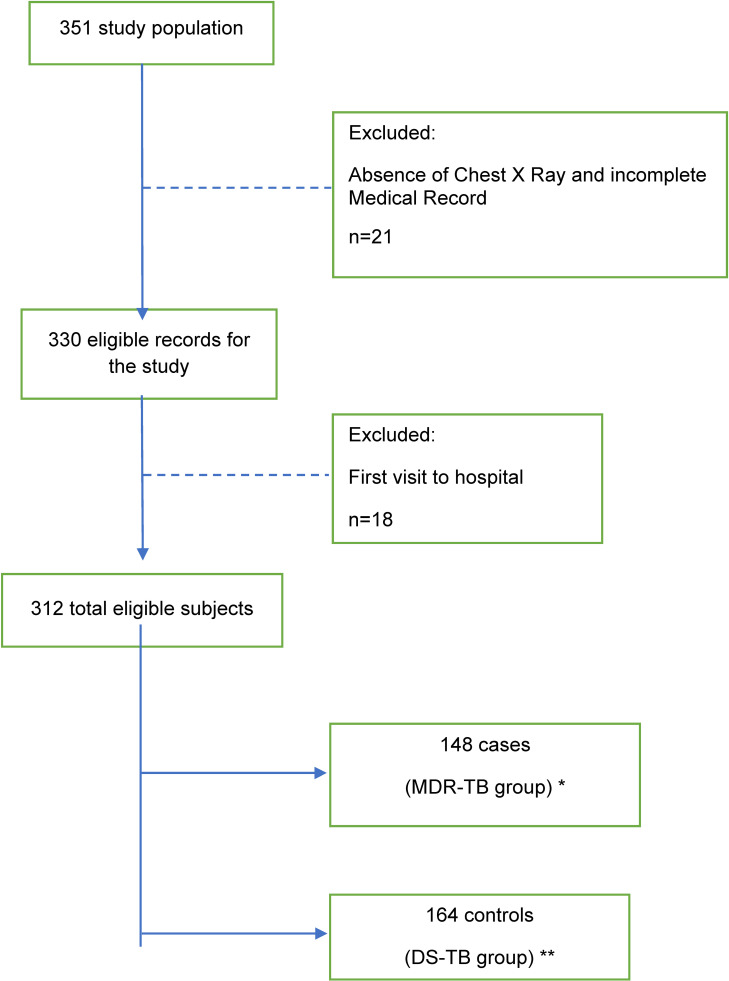
Flowchart of Study Participants. *MDR = Multidrug-resistant tuberculosis **DS-TB = Drug sensitive tuberculosis

A total of 351 patients were interviewed between March and April 2022. We excluded 21 patients due to absence of a chest X-ray and incomplete medical records. In addition, we excluded eighteen patients, those for whom we could not determine the role of PMO during treatment because they made a first time visit to the hospital. Finally, we have included 312 patients: 148 MDR-TB patients (case) and 164 DS-TB patients (control) (Fig. 1).

Most of the patients were males (68.6%), married (80.1%), and with low income (76.0%). Table 1 compares information between cases and controls. The mean age of cases and controls was similar, 41.9 (± 11.2) years vs 41.7 (± 12.1) years. Compared with the controls, a higher proportion of patients in cases reported low income, unmarried or divorced, abuse alcohol, relapsed, treatment failure or dropping out from previous treatment, suffering drug side effects, and longer traveling time to a hospital. A higher proportion of patients in cases reported their PMOs were less supportive, 44.6% vs. 26.2%. The majority of PMOs were from family members (81.8% in cases and 85.9% in controls [Supplementary Fig. 1]). A similar proportion of both groups reported the possibility of occupational silica exposure (27.1% vs 25.0%).

**Table 1:** Characteristic of Study Participants

**Variables**	**MDR-TB** **(n = 148)**	**DS-TB** **(n = 164)**	**p Value**
Age, years (mean, SD)	41.9 ± 11.2	41.7 ± 12.1	0.843
Categories of age (n, %)			0.942
<30 years (n, %)	24 (16.2)	31 (18.9)	
30–39 years	44 (29.7)	45 (27.5)	
40–49 years	39 (26.4)	44 (26.8)	
50–59 years	31 (20.9)	31 (18.9)	
≥60 years	10 (6.8)	13 (7.9)	
Sex (n, %)			0.180
Male	107 (72.3)	107 (65.2)	
Female	41 (27.7)	57 (34.8)	
Education (n, %)			0.473
Just literate/below elementary	11 (7.4)	19 (11.6)	
Elementary	45 (30.4)	43 (26.2)	
Junior High School	26 (17.6)	29 (17.7)	
Senior High School	55 (37.2)	63 (38.4)	
Bachelor	11 (7.4)	8 (4.9)	
Postgraduate	0 (0)	2 (1.2)	
Income (n, %)			0.023
<Regional Minimum Wage	121 (81.8)	116 (70.7)	
>Regional Minimum Wage	27 (18.2)	48 (29.3)	
Marriage (n, %)			0.010
Single	20 (13.5)	16 (9.7)	
Married	109 (73.7)	141 (86.0)	
Divorced or widowed	19 (12.8)	7 (4.3)	
Weight (mean, SD)	47.2 ± 8.9	48.9 ± 8.5	0.084
Height (mean, SD)	160.4 ± 9.4	160.4 ± 8.7	0.944
BMI kg/m2 (mean, SD)	18.4 ± 3.5	19.0 ± 3.0	0.032
BMI categories (n, %)			0.123
<18.5	84 (56.8)	75 (45.7)	
18.5–22.49	49 (33.1)	72 (43.9)	
>23	15 (10.1)	17 (10.4)	
Smoking (n, %)			0.213
Yes	102 (68.9)	102 (62.2)	
No	46 (31.1)	62 (37.8)	
Smoke per years (mean, SD)	9.3 ± 13.4	9.3 ± 12.7	0.979
Categories of smoke per years (n, %)			0.223
0 pack years	46 (31.1)	63 (38.2)	
<10 pack years	59 (39.8)	51 (30.9)	
≥10 pack-years	43 (29.1)	51 (30.9)	
Alcohol consumption (n, %)			0.002
Yes	50 (33.8)	30 (18.3)	
No	98 (66.2)	134 (81.7)	
History of treatment (n, %)			<0.001
New	25 (16.9)	117 (71.3)	
Relapse	70 (47.3)	26 (15.9)	
Failed	33 (22.3)	7 (4.3)	
Drop Out	17 (11.5)	11 (6.7)	
Unknown	3 (2.0)	3 (1.8)	
Contact history (n, %)			0.728
Yes	20 (13.5)	20 (12.2)	
No	128 (86.5)	144 (87.8)	
Drug side effect (n, %)			<0.001
Yes	133 (89.9)	59 (35.9)	
No	15 (10.1)	105 (64.1)	
Travelling time (n, %)			<0.001
<1 hour	11 (7.4)	52 (31.7)	
1–3 hours	111 (75.0)	93 (56.7)	
>3 hours	26 (17.6)	19 (11.6)	
Role of PMO (n, %)			0.001
Supportive	82 (55.4)	121 (73.8)	
Less supportive	66 (44.6)	43 (26.2)	
Silica exposure (n, %)			0.683
No	108 (72.9)	123 (75.0)	
Yes	40 (27.1)	41 (25.0)	
Duration of exposure (mean, SD)	3.9 ± 7.2	3.5 ± 7.8	0.639
Categories of duration exposure (n, %)			0.360
0 years (n, %)	101 (68.24)	118 (72.0)	
<10 years	23 (15.54)	21 (12.8)	
10–19 years	16 (10.81)	11 (6.7)	
≥20 years	8 (5.41)	14 (8.5)	
SilicoTB (n, %)			
Yes	8 (5.4)	9 (5.5)	0.974
No	140 (94.6)	155 (94.5)	

### Occupational characteristics

Of the reported occupations with possible silica exposure, most of the patients engaged in construction work (22.2% in cases and 19.8% in controls), agriculture/farming (7.4% in cases and 13.6% in controls), while mining and related milling was identified in 2.5% in cases and 4.9% in controls (Supplementary Fig. 2).

### Radiographic finding

**Fig. 2:**
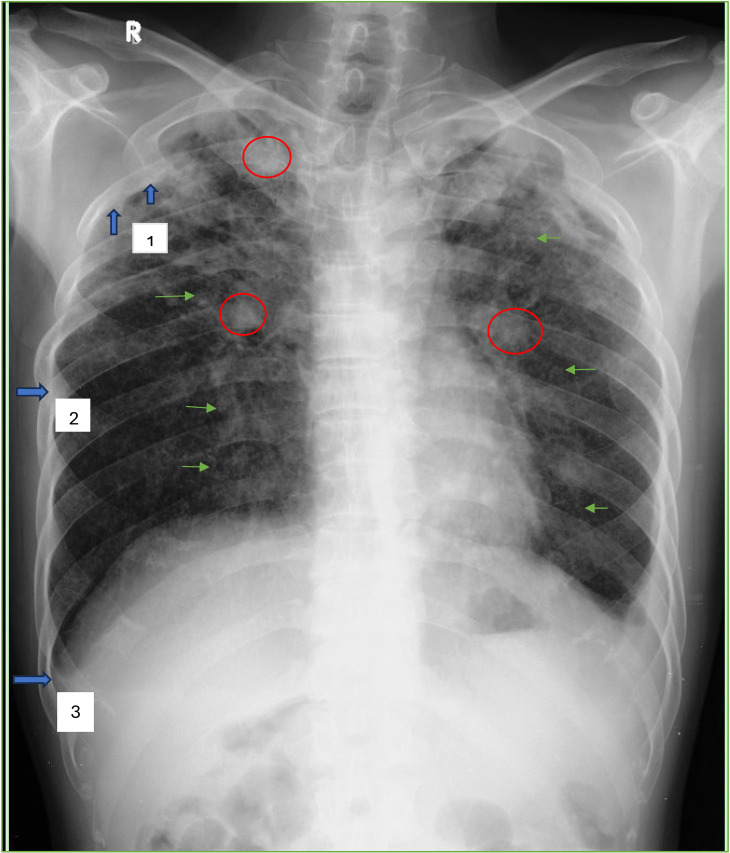
Definite category of silicosis with small, rounded opacities (green arrowheads) of the International Labour Office classification for profusion 3, predominant “q” size (1.5–3 mm), distributed diffusely in the upper, middle, and lower zones on both right and left lungs. There’s some large opacity (red circle) with size more than 1 cm in this case and also there’s some pleural thickening (blue arrowheads) in left apex (1), left in- profile (2) and in left costophrenic angle (3).

Radiographs consistent with silicosis were seen in 17 patients (5.4%), with about 8 people in definite categories and 9 people in the probable group (Table 2). Among positive cases, we observed that most were ILO profusion 3; in the upper and middle zones, the q shape predominated; 52.9% had large opacities and were dominated by size A. Pleural thickening was seen most prominently at the apex zone (Supplementary Fig. 3). The DS-TB group showed higher profusion as well as zones involvement, though it was not statistically significant. Examples of positive cases within definite categories are presented in Fig. 2.

**Table 2:** Chest Radiographic Patterns for Silicosis

**Radiographic Reading**	**MDR-TB** **n = 148**	**DS-TB** **n = 164**	**p value**
	n (%)		
Silicosis categories			0.029
Definite	2 (1.4)	6 (3.7)	
Probable	6 (4.1)	3 (1.8)	
Possible	24 (16.2)	12 (7.3)	
None	116 (78.3)	143 (87.2)	
	Subgroup of 17 patients with Radiographic silicosis		
	n = 8	n = 9	
Small Opacities			
Profusion			0.200
1	1 (12.5)	0 (0)	
2	4 (50.0)	2 (22.2)	
3	3 (37.5)	7 (77.8)	
Predominant size			
Small Rounded Opacity			0.187
p	0 (0)	3 (33.3)	
q	6 (75.0)	4 (44.5)	
r	2 (25.0)	2 (22.2)	
Small Irregular Opacity			NA
s	0 (0)	0 (0)	
t	0 (0)	0 (0)	
u	0 (0)	0 (0)	
Large Opacities			0.656
None	4 (50.0)	4 (44.5)	
A	2 (25.0)	4 (44.5)	
B	1 (12.5)	1 (11.1)	
C	1 (12.5)	0 (0)	
Zone Involvement^a^			0.164
4 zones	2 (25.0)	1 (11.1)	
5 zones	2 (25.0)	0 (0)	
6 zones	4 (50.0)	8 (88.9)	
	Subgroup of pleural thickening involvement		
Pleural thickening			
Lung apex^b^	7 (87.5)	6 (66.7)	0.312
Chest wall^c^	1 (12.5)	2 (22.2)	0.600
Other sites^d^	2 (25.0)	4 (44.5)	0.402

^a)^Zone involvement: The zones in the lung where opacities are visible are recorded. The lung field is split into six zones which are upper, middle, and lower in both right and left lung.

^b)^Lung apex of pleural involvement: the appearance of thickness in apex zone for both right and left lung

^c)^Chest wall of pleural involvement: the appearance of thickness on the chest wall **(**in-profile or face-on) for both in right and left lung

^d)^Other sites of pleural involvement: the appearance of thickness on costophrenic angle, diaphragm and other sites (the mediastinal pleura, in the para-spinal or para-cardiac locations) for both in right and left lung

### Risk factors associated with developing MDR-TB

Table 3 reports the crude and adjusted odds ratios for developing MDR-TB. It reveals that having supportive PMO reduced the risk of developing MDR-TB (aOR: 0.31, 95% CI: 0.15–0.62). Other factors that were significantly associated with the risk of developing MDR-TB were marital status (divorced or widowed) (aOR: 3.45, 95% CI: 1.26–9.46), alcohol consumption (aOR: 1.97, 95% CI: 1.05–3.69), low income (aOR: 2.28, 95% CI: 1.27–4.09), longer traveling time to hospital (aOR: 7.28, 95% CI: 2.92–18.19), unsuccessful previous treatment (relapse, treatment failure, dropping out) and encountering drug side effects (aOR: 17.69, 95% CI: 8.33–37.57).

**Table 3:** Crude and adjusted Odd Ratio with having MDR-TB

**Variables**	**Crude Odd Ratio** **(95% CI)**	**Adjusted Odd Ratio** **(95% CI)**
Sex, female	0.72 (0.44–1.16)	0.71 (0.31–1.64)
BMI categories (n, %)		
<18.5	1.65 (1.02–2.65)	1.48 (0.75–2.92)
18.5–22.49	Ref	Ref
>23	1.29 (0.59–2.84)	1.28 (0.40–4.12)
Income, <Regional Minimum Wage	1.85 (1.09–3.17)	2.28 (1.27–4.09)
Marital status		
Single	1.62 (0.80–3.27)	1.17 (0.51–2.68)
Married	Ref	Ref
Divorced or widowed	3.51 (1.42–8.65)	3.45 (1.26–9.46)
Smoke per years		
0 pack years	Ref	Ref
<10 pack-years	1.56 (0.91–2.66)	0.88 (0.37–2.09)
≥10 pack-years	1.14 (0.65–1.98)	0.61 (0.24–1.54)
Alcohol consumption, yes	2.28 (1.35–3.84)	1.97 (1.05–3.69)
Travelling time		
<1 hour	Ref	Ref
1–3 hours	5.64 (2.78–11.43)	5.66 (2.73–11.77)
>3 hours	6.47 (2.69–15.58)	7.28 (2.92–18.19)
Role of PMO, supportive	0.44 (0.27–0.71)	0.31 (0.15–0.62)
History of treatment		
New	Ref	Ref
Relaps	12.6 (6.75–23.51)	13.17 (6.08–28.48)
Failed	22.06 (8.76–55.52)	20.08 (6.76–59.67)
Drop Out	7.23 (3.02–17.31)	8.69 (2.79–26.98)
Unknown	4.68 (0.89–24.55)	4.53 (0.76–26.84)
Drug side effect, yes	15.78 (8.47–29.39)	17.69 (8.33–37.57)

## Discussion

In this hospital-based case-control study, we attempted to determine the association of silicosis and the role of a PMO in developing MDR-TB by surveying and reading chest x-rays of all subjects. Findings indicate that the presence of supportive PMO reduces the risk of developing MDR-TB, but silicosis showed no significant association. Nevertheless, we found that 17 cases (5.4%) had silico-TB in this study, although the small number of positive cases did not allow us the ability to detect statistically significant differences between the groups.

In both silicosis and TB, we had similar opacities, which were small rounded opacities, thus making this study unique. Differences in the characteristic of these opacities were whether they were diffuse or focal, as well as the continuity of their location. The presence of focal small rounded opacities may indicate active TB, as evidenced by the tree-in-bud appearance on a CT [21].

Neither a job history of silica exposure nor radiographic silicosis increased the likelihood of developing MDR-TB. Radiologic evidence of silicosis is less prevalent with less than seven years of exposure unless the environment is heavily contaminated [22]. In our study, the mean duration of silica exposure between two groups were similar (3.9 ± 7.2 years vs 3.5 ± 7.8 years) and the dust concentration was not disclosed, which could have varied depending on the job.

In the present study, the prevalence of 5.4% with silico-TB is lower than other studies reported in Transkei (28.4%) [23], Basotho (25.7%) [24] and India (7.4%) [25]. However, the prevalence shown here is specific for TB-treating hospitals while the others were conducted with mining workers; thus, the best approach to see radiographic silicosis is probably through the Medical Check Up (MCU) for workers who have been exposed to silica. Since we do not have regulations for screening and surveillance for pneumoconiosis among workers, this study could serve as a trigger for authorities to enact legislation on the subject.

Although the effectiveness of DOT has been questioned for a long time [13], this study shows that the existence of a supporting PMO is crucial in preventing TB patients from developing MDR-TB. In our study, most of the PMOs were family members. According to the WHO, health-care workers and trained lay providers were the preferred DOT provider over family members [26]. However, past studies reported positive outcomes in TB treatment where family members serve as DOT providers [27, 28].

We observed that patients in the MDR-TB group reported that their PMOs were least supportive regarding information about regular treatment, while a study by Murtiwi found that *66.6%* subjects are never reminded to take the drug [17]. In our study, among both the MDR-TB and DS-TB groups, how to deal with drug side effects was also not well communicated to PMOs (Supplementary Table 1). Supervision by the PMO is still difficult to standardize because the characteristics and performance largely vary among PMOs. This is understandable considering the limitation of knowledge regarding medications. As a result, if family members are providing DOT, they must be carefully identified and trained, and additional supervision of local supporters or health-care workers may still be required.

This study also identified several variables as risk factors for MDR-TB, such as treatment side effects. Our investigation found that 89.9% of MDR-TB patients had at least one adverse event, which was greater than the 57.3% reported in a meta-analysis [29]. They also reported more serious and diverse problems during the interview. These pharmacological side effects have been recognized as the cause of recurrence and failure to finish therapy. Numerous studies have established that prior medication use is the most important predictor of MDR [8, 9, 11] and our investigation confirmed this. Several reasons have been linked to high recurrence rates in Indonesia, including fear of side effects, poor counseling and support, fraudulent therapies on the internet, and stigma and prejudice from family or community [1]. Alcohol use is also a risk factor in this study. Alcohol has a deleterious effect on the immune system, increasing vulnerability to other diseases, re-infection, treatment failure, or altering the pharmacokinetics of tuberculosis medications [30]. Furthermore, we identified divorce as a risk factor for MDR-TB. A study reported that lack of social and family support predisposed TB patients to stop or interrupt the treatment [31]. We also discovered that longer travel times to the hospital and poor income were risk factors for acquiring MDR-TB. The lengthy travel time of respondents suggests that access to health care is far and time-consuming, which could be one of the reasons patients do not seek routine treatment. Although TB treatment is free of charge, yet uncovered travel and food/nutritional supplement costs increase the cost burden for patients. A study by Fuady revealed that in Indonesia, the median (IQR) of total costs households pay is 133 USD for DS-TB and 2,804 USD for MDR-TB. As TB needs long treatment periods and impairs health, patients lose jobs or income, which increases the expenditures and treatment adherence barriers [32].

In Indonesia, many workers are likely exposed to silica. Patients with TB and populations at high occupational risk should undergo active case detection for assessing silicosis by performing annual chest radiographies. Training to increase the ability to read chest radiographs for pneumoconiosis using the ILO Classification should be conducted for physicians, particularly those working in the national tuberculosis control program. To strengthen the understanding of the relationship between silica and MDR-TB in Indonesia, we recommend that future studies increase sample size, diversity and should broaden its geographical scope by incorporating more locations in Indonesia, particularly the area with significant silica exposure in industries such as Borneo Island, which has abundant coal and other mining activities, to enhance generalizability across different demographics and occupations.

The findings of this study might highlight the critical functions of family members. They should play a larger role as supervisors in Indonesia’s healthcare system. Families should get involved starting from planning to implementation and to evaluation of the TB response in all areas of TB care and prevention such as case finding, contact investigation and treatment support. The need for support is important when patients are stigmatized in the workplace or community [33, 34].

This study identified significant gaps in knowledge among family members serving as PMOs. Thus, healthcare institutions could implement educational program featuring a standardized curriculum to enhance their understanding of illnesses, treatments, and responsibilities, with emphasize communication skills and empathy to foster patient adherence and trust. This may result in the development of more organized training programs for family members serving as drug supervisors.

Our study had some limitations. First, we only evaluated patients from a single TB referral hospital, which limits the extrapolation of our findings to the entire population of Indonesian workers. Second, some of the variables were self-reported, including information on PMO and job history; we cannot exclude misclassification or recall bias. Since Indonesia lacks data on occupational exposure, we employed an approach to determine occupational silica exposure using the IndSIC classification system. Third, we have no quantitatively measured data for silica exposure. Despite these limitations, we believe our study provides crucial information about the importance of a PMO’s role in the management of TB patients and this is also the first study regarding the association between silicosis and MDR-TB in Indonesia.

## Conclusion

A supportive drug supervisor can serve as a preventive healthcare measure to protect TB patients from developing MDR-TB. Nevertheless, support from healthcare professionals and communities is still required alongside the assistance provided by family members delivering DOT. Although this study indicates that the development MDR-TB was not substantially affected by silicosis, we have demonstrated a prevalence of silicosis as evidenced by chest radiography. Given that this is the first documented report of silicosis prevalence in TB patients in Indonesia, we are emphasizing the importance of dust control, occupational hygiene, and health screening for high-risk population. The collaboration of employers, healthcare sectors and policymakers is also essential to ensure a safer work environment and better health outcomes.

## Supplementary information

**Additional file 1:** **Supplementary Table 1** Distribution of Answers Based on Question Items in the PMO’s Questionnaire. **Supplementary Table 2** The Terms of the 2011 ILO International Classification. **Supplementary Figure 1** Percentage of Participants’ PMO between Multidrug-resistant tuberculosis (MDR-TB) and Drug sensitive (DS-TB) groups. **Supplementary Figure 2** Distribution of occupations with possible exposure to silica. **Supplementary Figure 3** Percentage of Pleural Thickening.

## Declarations

### Availability of data and material

All data are incorporated into the article and its online supplementary material.
