# Supplementary material for: Influences of radiographic silicosis and drug supervisor on the development of multi drug resistant-tuberculosis in West Java, Indonesia
Source: Environ Health Prev Med. 2025 Mar 20;30:20. doi: 10.1265/ehpm.24-00169 (PMC11955800; doi:10.1265/ehpm.24-00169)
Supplement: Supplementary file 1 — Additional file 1: Supplementary Table 1 Distribution of Answers Based on Question Items in the PMO’s Questionnaire. Supplementary Table 2 The Terms of the 2011 ILO International Classification. Supplementary Figure 1 Percentage of Participants’ PMO between Multidrug-resistant tuberculosis (MDR-TB) and Drug sensitive (DS-TB) groups. Supplementary Figure 2 Distribution of occupations with possible exposure to silica. Supplementary Figure 3 Percentage of Pleural Thickening. [file ehpm-30-020-s001.docx]

**Supplementary Table 1** Distribution of Answers Based on Question Items in the PMO’s Questionnaire

| **No** | **Questions** | **Yes (%)** | | |
| --- | --- | --- | --- | --- |
|  |  | Total  (n=312) | MDR-TB  (n=148) | DS-TB  (n=164) |
| Q1 | Do you know who is in charge of your PMO? | 258 (82.7) | 122 (82.4) | 136 (82.9) |
| Q2 | Did anyone remind you to swallow your medication every day? | 236 (75.6) | 113 (76.4) | 123 (75.0) |
| Q3 | Did PMO constantly remind you to take your medication on a daily basis? | 249 (79.8) | 120 (81.1) | 129 (78.7) |
| Q4 | Are you constantly reminded to recheck the sputum at the appointed time? | 228 (73.1) | 114 (77.0) | 114 (69.5) |
| Q5 | Did the PMO educate other family members about tuberculosis symptoms? | 141 (45.2) | 56 (37.8) | 85 (51.8) |
| Q6 | Did PMO recommend that a family member visit the healthcare unit if they have had a cough for more than three weeks? | 160 (51.3) | 62 (41.8) | 98 (59.8) |
| Q7 | Have you ever been informed by PMO that tuberculosis is not a hereditary disease or a curse? | 127 (40.7) | 57 (38.5) | 70 (42.7) |
| Q8 | Did your PMO ever inform you that tuberculosis can be treated with regular treatment? | 214 (68.6) | 85 (57.4) | 129 (78.7) |
| Q9 | Did the PMO provide counseling on the importance of regular treatment? | 213 (68.3) | 86 (58.1) | 127 (77.4) |
| Q10 | Do you trust in PMO? | 256 (82.1) | 121 (81.7) | 135 (82.3) |
| Q11 | Did the PMO provide information about the risks of not taking medication regularly? | 200 (64.1) | 83 (56.1) | 117 (71.3) |
| Q12 | Did the PMO provide education on how TB is transmitted? | 154 (49.4) | 71 (47.9) | 83 (50.6) |
| Q13 | Did the PMO inform you about the adverse effects of drugs you've taken? | 134 (42.9) | 69 (46.6) | 65 (39.6) |
| Q14 | Did your PMO provide you instructions if you experience side effects? | 120 (38.5) | 57 (38.5) | 63 (38.4) |
| Q15 | Did your PMO inform you about how to take TB treatment regularly? | 193 (61.9) | 75 (50.7) | 118 (71.9) |

**Supplementary Table 2** The Terms of the 2011 ILO International Classification

| Terms | Codes | Explanation |
| --- | --- | --- |
| Profusion | 1,2,3 | The concentration of small opacities per zone of lung, calculated by comparing the patient's chest radiograph to standard radiographs published by the ILO.  Categories 1, 2 and 3 = Assessment of the number of small opacities per unit area or zone of lung and increasing profusion of small opacities. |
| Small rounded opacities | p,q,r | Well-defined opacities or nodules ranging with a diameter of up to 10 mm, ranging from barely visible.  p= diameter up to about 1.5 mm q= diameter exceeding about 1.5 mm and up to about 3 mm r = diameter exceeding about 3 mm and up to about 10 mm |
| Small irregular opacities | s,t,u | Linear, reticular or reticulonodular opacities. s = width up to 1.5 mm t = width exceeding about 1.5 mm and up to about 3 mm u= width exceeding about 3 mm and up to about 10 mm |
| Large opacities | A, B, C | Refers to progressive massive fibrosis. An opacity with a longest dimension longer than 10 mm is considered as large opacity.  A= one larger opacity with a diameter higher than 1 cm and up to 5 cm, or many opacities with, the sum of whose greatest diameters less than 5 cm. B= one or more opacities that are greater or more numerous than those in category A, but their total area is still less than that of the right upper lung zone.  C= one or more opacities whose combined area larger than the right upper lung zone. |
| Zone involvement | -Upper right zone  - Upper left zone  - Middle right zone  - Middle left zone  - Lower right zone  -Lower left zone | The zones in the lung where opacities are visible are recorded. Each lung field is split into three zones (upper, middle, and lower) by horizontal lines drawn at about one-third and two-thirds of the vertical distance between the lung apices and the diaphragmatic domes. |
| Pleural thickening | -Apex zone  - In profile zone  - Face on zone  - Costophrenic angle  - Diaphragm  - Para-spinal  -Para-cardiac | In certain locations the appearance of thickness indicates pleural involvement. The measurement of the pleural thickening along the lateral chest wall is the distance from the parenchymal-pleural boundary to the inner line of the chest wall.  Pleural thickening may be seen on apex, on the chest wall (in-profile or face-on) separately for the right and left sides, and at other sites include costophrenic angle, diaphragm, the mediastinal pleura in the para-spinal or para-cardiac locations. |

**Supplementary Figure 1** Percentage of Participants’ PMO between Multidrug-resistant tuberculosis (MDR-TB) and Drug sensitive (DS-TB) groups.

*PMO=Pengawas Menelan Obat (Drug Supervisor for TB Patients)

**Supplementary Figure 2** Distribution of occupations with possible exposure to silica.

**Supplementary Figure 3** Percentage of Pleural Thickening

*CPA= Costophrenic angle

** Others= Mediastinal pleura in the para-spinal or para-cardiac locations
